# Clinicopathologic and prognostic factors of patients with T3/T4 colorectal signet ring cell carcinoma: a population-based study

**DOI:** 10.1007/s00432-023-04880-2

**Published:** 2023-05-28

**Authors:** Fan Zhang, Boqi Xu, Yao Peng, Zhongqi Mao

**Affiliations:** Department of General Surgery, The First Affiliated Hospital of Soochow University, Suzhou, China

**Keywords:** Colorectal cancer, Signet ring cell carcinoma, Survival, Nomogram, SEER database

## Abstract

**Background:**

To evaluate cancer-specific survival (CSS) and construct a nomogram to predict the CSS of patients with colorectal signet ring cell carcinoma (SRCC).

**Methods:**

The data for patients with colorectal SRCC from 2000 to 2019 was identified from Surveillance, Epidemiology, and End Results (SEER) database. Propensity Score Matching (PSM) was used to minimize bias between SRCC and adenocarcinoma patients. Kaplan–Meier method and log-rank test were used to estimate the CSS. A nomogram was constructed based on the independent prognostic factors identified by univariate and multivariate Cox proportional hazards regression analyses. The model was evaluated by receiver operating characteristic (ROC) curves and calibration plots.

**Results:**

Poor CSS was more common in patients with colorectal SRCC, especially in patients with T4/N2 stage, tumor size > 80 mm, grade III-IV, and chemotherapy. Age, T/N stage, and tumor size > 80 mm were identified as independent prognostic indicators. And a prognostic nomogram was constructed and validated as an accurate model for the CSS of patients with colorectal SRCC by ROC curves and calibration plots.

**Conclusion:**

Patients with colorectal SRCC have a poor prognosis. And the nomogram was expected to be effective in predicting the survival of patients with colorectal SRCC.

## Introduction

Colorectal cancer (CRC) is the third most commonly diagnosed malignancy based on Cancer statistics in 2023 in the United States nationally and for each state. Approximately 153,020 new CRC cases will be analyzed, and 52,550 patients will suffer cancer deaths in 2023 (Siegel et al. 2023). Signet ring cell carcinoma (SRCC) with abundant intracytoplasmic mucin is commonly diagnosed in gastric cancer, but it is a rare pathological subtype of CRC. Only 1% of all pathological types of rectal cancer cases were diagnosed with SRCC (Benesch and Mathieson 2020). SRCC was associated with more aggressive behaviors, a higher rate of lymph node involvement, and a worse prognosis, especially in advanced colorectal cancer (Song et al. 2019). There are several consistent findings focused on the clinicopathological characteristics of patients with early-onset colorectal cancer (patients < 50 years old), which demonstrated that the presence of signet-ring cells was more frequent in the younger age patients (Vuik et al. 2021; Zaborowski et al. 2021). As a result, it is essential to evaluate the prognosis of SRCC patients, even with low incidence.

Although previous studies have demonstrated that the survival rates of colorectal SRCC were associated with the primary tumor site, stages of the tumor, and lymphatic invasion (Kim et al. 2021; Liang et al. 2018), appropriate prediction guidelines for survival rates of SRCC patients are currently lacking due to the low incidence of SRCC and the lack of clinical data. The prognosis of SRCC is still controversial, and it has even been reported that the prognosis of early SRCC is better than that of early adenocarcinoma (Chon et al. 2017).

Hence, we reevaluated the survival rates and constructed a predictive model for survival rates of advanced colorectal SRCC based on the Surveillance, Epidemiology, and End Results (SEER) database.

## Materials and methods

### Patients

Clinicopathological data of patients diagnosed with colorectal SRCC between 2000 and 2019 were extracted from the program SEER*Stat (Version 8.4.0.1) based on Incidence-SEER Research Plus Data, 17 Registries, Nov 2021 Sub (2000–2019). Inclusion criteria included: (1) Pathological stage T3-4/N0-2/M0 colorectal cancer; (2) Patients undergoing surgery for colorectal cancer; (3) Patients died due to colorectal cancer; (4) TNM stage, histological type, age, grade, tumor size, and primary tumor site were available. Finally, 1051 SRCC patients were recruited for the study.

### Search strategy and patient cohort

Firstly, patients with primary tumor site labeled as C18.2, C18.3, C18.4, C18.5, C18.6, C18.7, C18.9, C19.9, and C20.9 were included in the study. Secondly, the following variables were collected from the database: age, sex, race (White, Black, Other), year of diagnosis, primary tumor site, tumor grade (Grade I, Grade II, Grade III, Grade IV), tumor histological type (Adenocarcinoma, Signet ring cell carcinoma), TNM stage, treatment options (Surgery, Chemotherapy), tumor size, tumor behavior, survival state, and survival months. The primary tumor site labeled by the ICD-0–3 site code was divided into two groups: colon and rectum. Tumor grades were classified as grade I-II and grade III-IV. Patients with unknown tumor grades would be excluded from this dataset. Tumor histological types of 8140/3: adenocarcinoma and 8490/3: signet ring cell carcinoma were included in the study according to ICD-0–3 Hist/behave. T and N stages were categorized by the 6th edition of the American Joint Committee on Cancer (AJCC) tumor-node-metastasis (TNM) staging system between 2004 and 2015. Only patients who had undergone colorectal cancer surgery were included in the study.

### Propensity score-matching (PSM)

To eliminate the bias and confounding factors between adenocarcinoma and SRCC groups, we performed the Propensity Score-Matching (PSM). The propensity score model included age, sex, race, T stage, N stage, primary tumor site, tumor grade, size, and chemotherapy. The logistic regression model was used to calculate propensity scores. All adenocarcinoma patients were matched with SRCC patients based on propensity scores in 1:1. Before and after PSM, clinicopathological variables were compared using *χ*^2^ test to assess the feasibility of PSM.

### Statistical analysis

R software version 4.4.2 (Institute for Statistics and Mathematics, Vienna, Austria; https://www.r-project.org/) was used for statistical analysis. After PSM, cancer-specific survival (CSS) was estimated using the Kaplan–Meier (KM) method and log-rank tests. We included tumor histological type, chemotherapy, tumor grade, N stage, T stage, and tumor size to compare the survival rates of the tumor. In the SRCC group, univariate and multivariate Cox proportional hazards regression analyses were employed to evaluate the independent prognostic indicators of CSS. Clinicopathological variables with *P* < 0.05 in univariate analysis were further performed in multivariate analysis. A novel prognostic nomogram was constructed based on clinicopathological variables with *P* < 0.05 in multivariate Cox analysis to predict the CSS of SRCC patients. In addition, the area under the curve (AUC) of the receiver operator characteristic (ROC) was calculated to assess the prediction efficiency of the model. Calibration curves were plotted to evaluate differences in survival rates between the nomogram model and observation groups at 12-, 24-, and 36-month after surgery.

## Results

### Patient characteristics and propensity score-matching

**Fig. 1:**
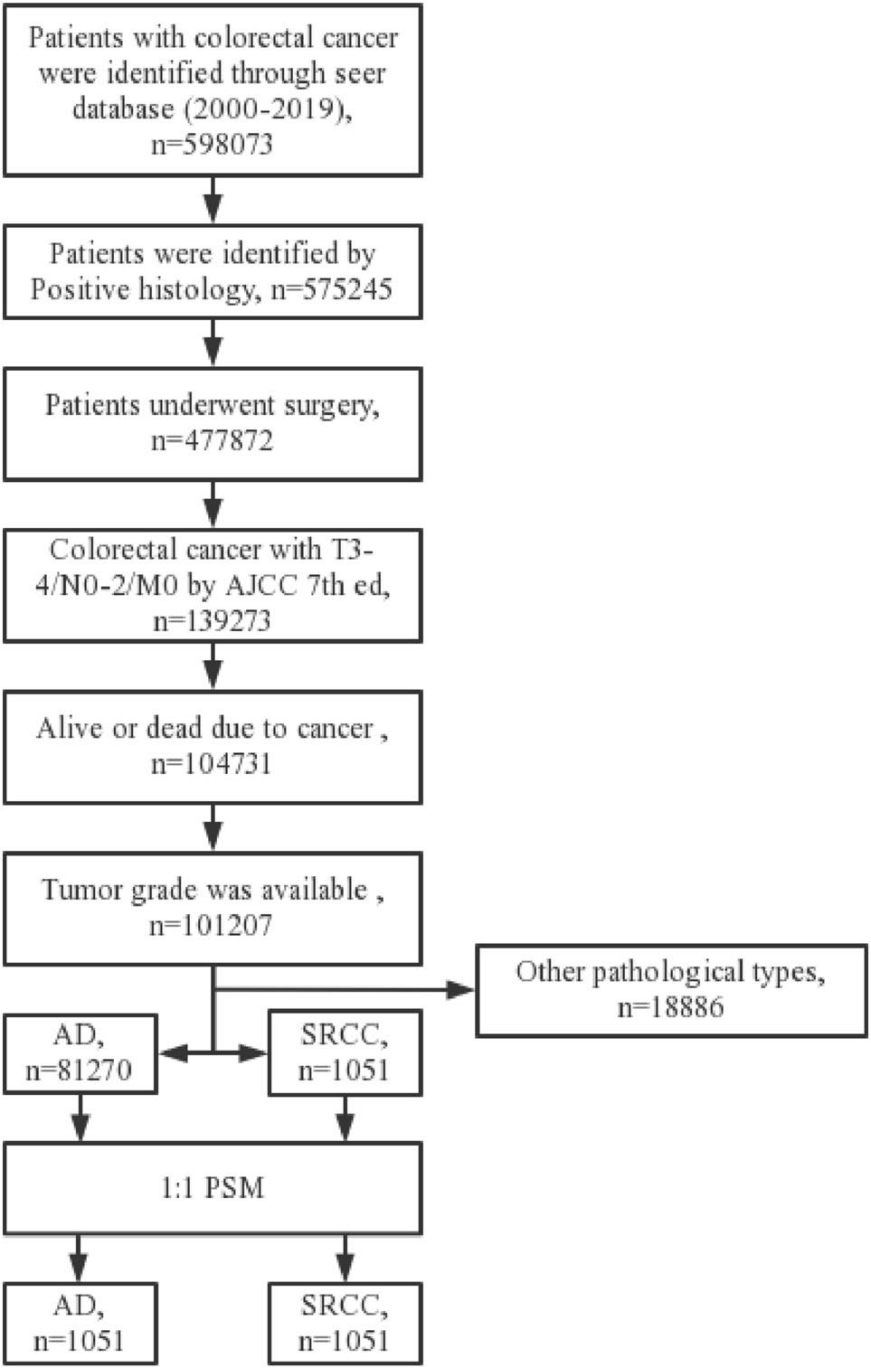
Flow chart depicting the patient selection process. *AD* Adenocarcinoma, *SRCC* signet ring cell carcinoma, *PSM* propensity score matching

**Fig. 2:**
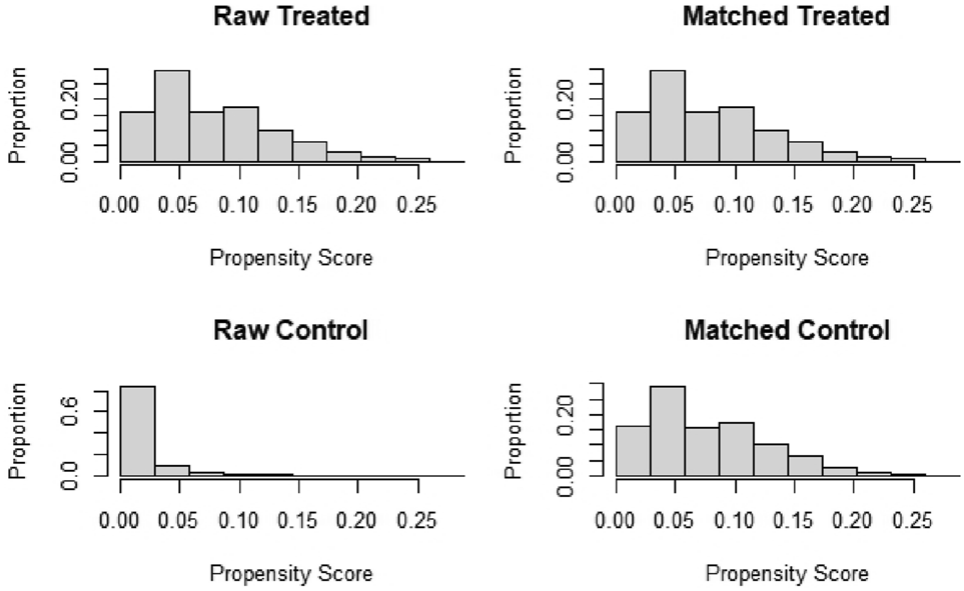
Propensity score distribution between patients with signet ring cell carcinoma (SRCC) and adenocarcinoma before and after propensity score matching

Table 1 shows the essential characteristics of patients before and after PSM. A total of 81,270 patients with adenocarcinoma and 1051 patients with SRCC were extracted from the seer database, respectively (Fig. 1). In patients with colorectal adenocarcinoma, 64,643 patients had primary tumor sites in the colon, and 16,627 patients had primary tumor sites in the rectum before PSM. And 855 patients had primary tumor sites in the colon, and 196 patients had primary tumor sites in the rectum in SRCC patients. Of these patients, 51.5% of adenocarcinoma and 59.6% of SRCC patients received postoperative chemotherap. Significant differences were present in the T stage, N stage, tumor grade, size, and chemotherapy (*P* < 0.001). After 1:1 PSM, 1051 patients remained in each group. There were no significant differences between groups for baseline characteristics. The propensity score distribution between adenocarcinoma and SRCC patients before and after PSM is shown in Fig. 2.

**Table 1:** Baseline characteristics before and after propensity score matching, showing statistical comparisons between colorectal adenocarcinoma and signet ring cell carcinoma (SRCC) groups (*χ*^2^ test)

	Pre-PSM				Post-PSM		
	AD (*N* = 812,705), *n*%		SRCC (*N* = 1051), *n*%	*P* value	AD (*N* = 1051), *n*%	SRCC (*N* = 1051), *n*%	*P* value
Age				0.830			0.892
< 60	29,702 (36.5%)		388 (36.9%)		384 (36.5%)	388 (36.9%)	
> = 60	51,568 (63.5%)		663 (63.1%)		667 (63.5%)	663 (63.1%)	
Sex				0.280			0.896
Female	38,672 (47.6%)		482 (45.9%)		486 (46.2%)	482 (45.9%)	
Male	42,598 (52.4%)		569 (54.1%)		565 (53.8%)	569 (54.1%)	
Race				0.127			0.489
Black	3635 (4.5%)		39 (3.7%)		30 (2.9%)	39 (3.7%)	
White	69,907 (86.0%)		927 (88.2%)		941 (89.5%)	927 (88.2%)	
Other	7728 (9.5%)		85 (8.1%)		80 (7.6%)	85 (8.1%)	
T stage				< 0.001			1.000
T3	68,009 (83.7%)		708 (67.4%)		709 (67.5%)	708 (67.4%)	
T4	13,261 (16.3%)		343 (32.6%)		342 (32.5%)	343 (32.6%)	
N stage				< 0.001			1.000
N0	40,383 (49.7%)		256 (24.4%)		256 (24.4%)	256 (24.4%)	
N1	26,534 (32.6%)		248 (23.6%)		248 (23.6%)	248 (23.6%)	
N2	14,353 (17.7%)		547 (52.0%)		547 (52.0%)	547 (52.0%)	
Tumor site				0.159			0.822
Colon	64,643 (79.5%)		855 (81.4%)		860 (81.8%)	855 (81.4%)	
Rectum	16,627 (20.5%)		196 (18.6%)		191 (18.2%)	196 (18.6%)	
Grade				< 0.001			0.986
Grade I	4489 (5.5%)	10 (1.0%)			11 (1.0%)	10 (1.0%)	
Grade II	61,170 (75.3%)	70 (6.7%)			69 (6.6%)	70 (6.7%)	
Grade III	13,885 (17.1%)	798 (75.9%)			793 (75.5%)	798 (75.9%)	
Grade IV	1726 (2.1%)	173 (16.5%)			178 (16.9%)	173 (16.5%)	
Tumor size				< 0.001			0.953
< 40	26,412 (32.5%)	198 (18.8%)			199 (18.9%)	198 (18.8%)	
40–80	44,430 (54.7%)	604 (57.5%)			609 (57.9%)	604 (57.5%)	
> 80	10,428 (12.8%)	249 (23.7%)			243 (23.1%)	249 (23.7%)	
Chemotherapy				< 0.001			0.894
No/unknown	39,437 (48.5%)	425 (40.4%)			429 (40.8%)	425 (40.4%)	
Yes	41,833 (51.5%)	626 (59.6%)			622 (59.2%)	626 (59.6%)	

*PSM* propensity score matching, *AD* adenocarcinoma, *SRCC* signet ring cell carcinoma

### Survival outcomes after propensity score-matching

**Fig. 3:**
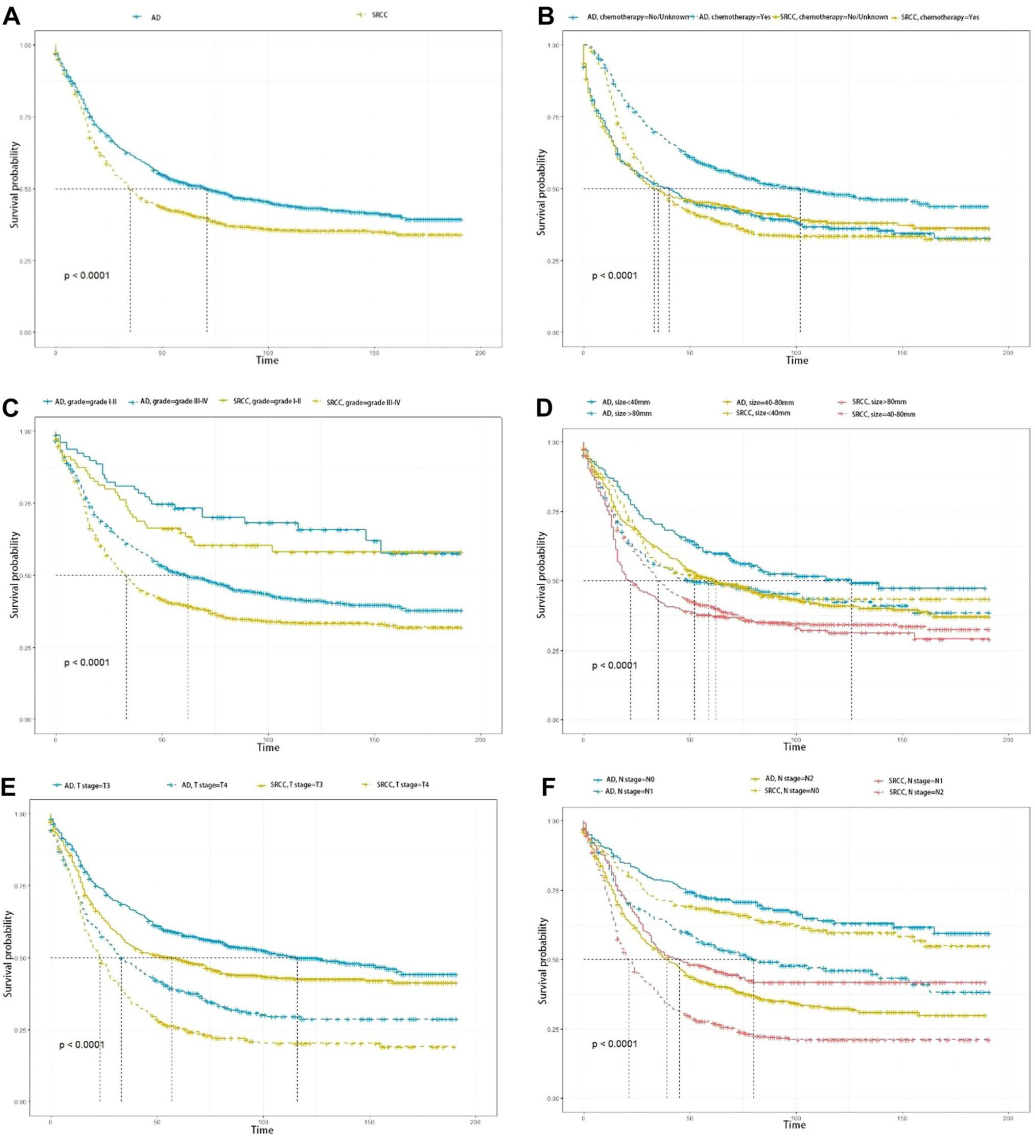
Kaplan–Meier (KM) survival curves comparing the cancer-specific survival (CSS) of patients with signet ring cell carcinoma (SRCC) and adenocarcinoma (AD). **A** pathological types; **B** chemotherapy; **C**)tumor grade; **D** tumor size; **E** T stage; **F** N stage

KM curves and log-rank tests were employed to evaluate CSS based on tumor histological type, chemotherapy, tumor grade, N stage, T stage, and tumor size (Fig. 3). The median survival time was 45 months (0–191 months). Of these, 656 SRCC and 566 adenocarcinoma patients died from colorectal cancer, respectively. The median CSS in SRCC patients was 31–40 months which was significantly shorter than adenocarcinoma patients (56–87 months) (log-rank test: *χ*^2^ = 19.5, *P* < 001). Regarding tumor grade I-II, T3 stage, N0/N1 stage, and < 40 mm tumor size, patients were presented with more prolonged survival (*P* < 0.001).

### Univariate and multivariate cox analyses for CSS of SRCC

Univariate and multivariate Cox analyses of SRCC patients are shown in Table 2. We included data on age, sex, race, T/N stage, tumor site, grade, tumor size, and chemotherapy in univariate Cox analysis. Age, sex, T stage, N stage, and tumor size were significantly identified according to univariate analysis (*P* < 0.05). Age (HR = 1.61, *P* < 0.001), T stage (HR = 1.46, *P* < 0.001), N stage (N1: HR = 1.69 *P* < 0.001; N2: HR = 2.99, *P* < 0.001), tumor size (40-80 mm: HR = 1.2, *P* = 0.089; > 80 mm: HR = 1.49, *P* = 0.001) were independent prognostic factors for CSS of SRCC patients according to multivariate analysis.

**Table 2:** Univariate and multivariate analyses of prognostic factors associated with cancer-specific survival of patients with SRCC

	Univariate analysis			Multivariate analysis		
	HR	95%CI	*P*	HR	95%CI	*P*
Age						
< 60	Reference			Reference		
> = 60	1.28	1.12–1.47	0.002	1.61	1.40–1.85	< 0.001
Sex						
Female	Reference			Reference		
Male	1.19	1.05–1.36	0.026	1.15	1.01–1.31	0.082
Race						
White	Reference					
Black	1.14	0.83–1.58	0.497			
Other	1.19	0.95–1.48	0.207			
T stage						
T3	Reference			Reference		
T4	1.78	1.56–2.03	< 0.001	1.46	1.28–1.67	< 0.001
N stage						
N0	Reference			Reference		
N1	1.74	1.40–2.16	< 0.001	1.69	1.36–2.10	< 0.001
N2	3.04	2.52–3.66	< 0.001	2.99	2.48–3.62	< 0.001
Tumor site						
Colon	Reference					
Rectum	1.18	1.01–1.38	0.091			
Grade						
Grade I	Reference					
Grade II	1.16	0.48–2.79	0.784			
Grade III	2.32	1.02–5.30	0.094			
Grade IV	2.54	1.10–5.87	0.067			
Tumor size						
< 40	Reference			Reference		
> 80	1.52	1.24–1.86	< 0.001	1.49	1.21–1.83	0.001
40–80	1.32	1.11–1.58	0.011	1.20	1.00–1.44	0.089
Chemotherapy						
No/unknown	Reference					
Yes	0.97	0.85–1.11	0.747			

### Development and validation of a prognostic nomogram

**Fig. 4:**
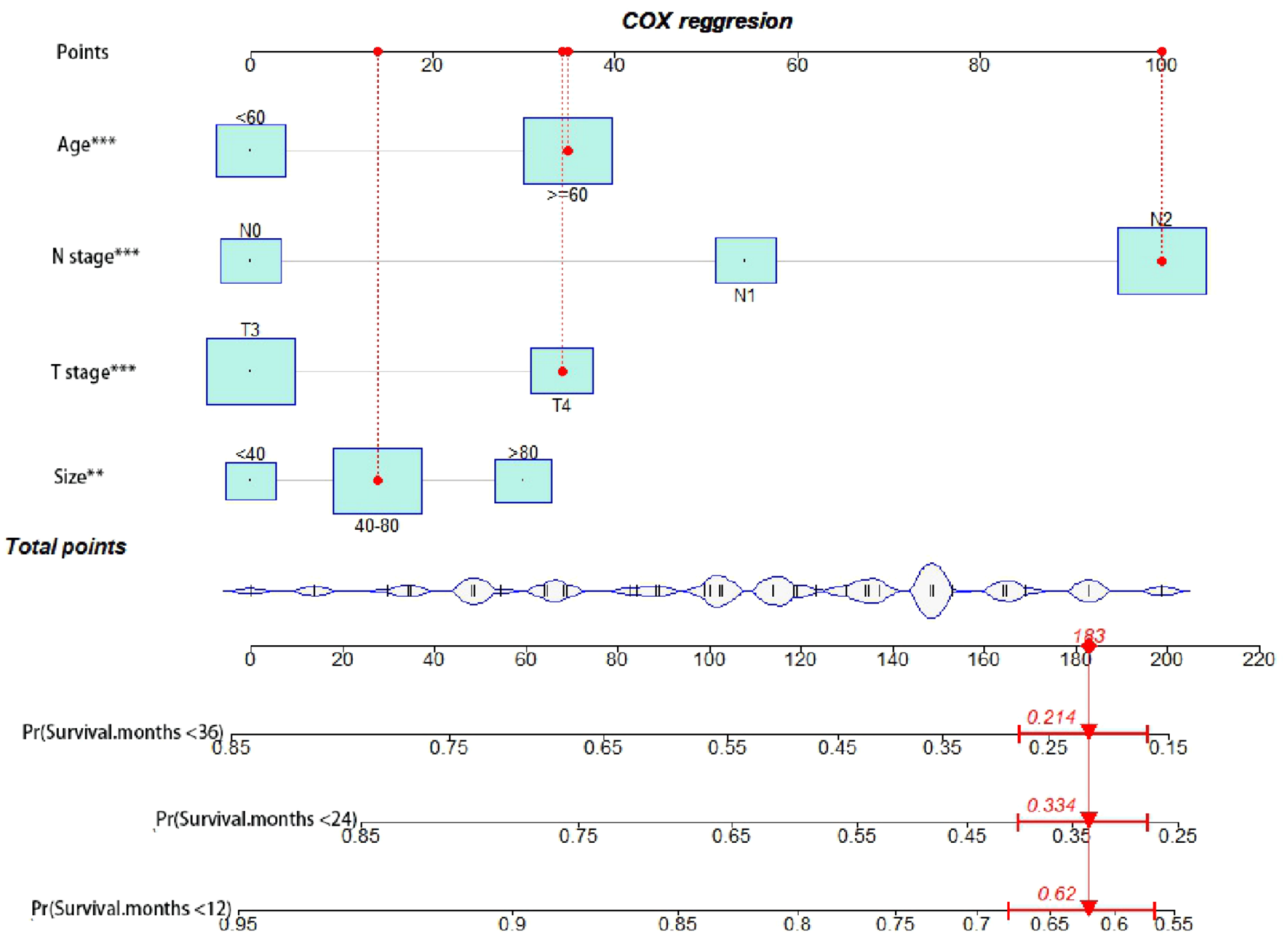
A prognostic nomogram predicting the cancer-specific survival (CSS) of patients with signet ring cell carcinoma (SRCC) and adenocarcinoma for the 12, 24, and 36 months

The nomogram can transform the prediction model results into readability and help clinicians assess the CSS of patients. A novel prognostic nomogram was constructed based on T stage, N stage, age, and tumor size to determine the 12-, 24-, and 36-month CSS of patients with SRCC, as shown in Fig. 4. The nomogram showed that the N stage had the most significant impact, and tumor size had the most negligible impact on the CSS.

**Fig. 5:**
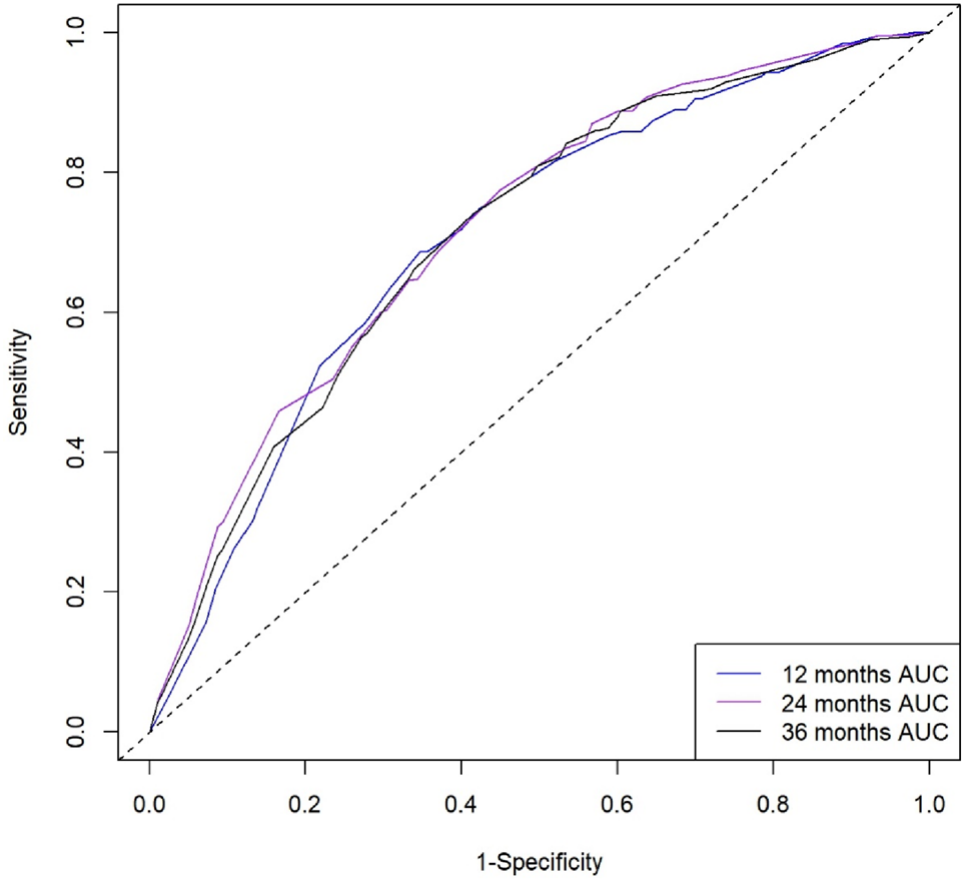
Time-dependent receiver operating characteristic (ROC) curves for the 12, 24, and 36 months

**Fig. 6:**
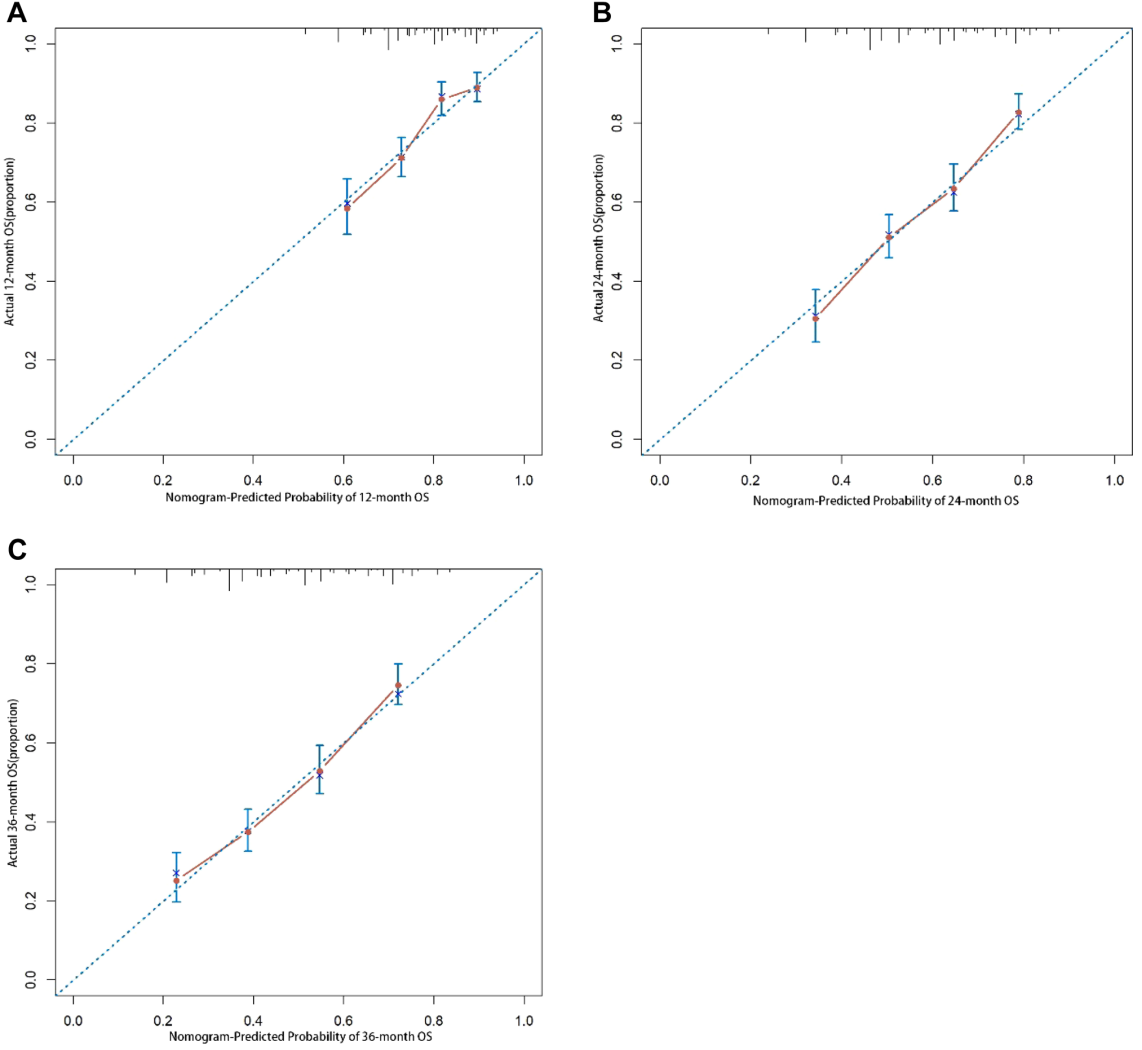
Calibration plots of 12- (**A**), 24- (**B**), and 36-month (**C**) overall survival (OS) for signet ring cell carcinoma (SRCC) patients

The C- indices value of SRCC patients for predicting 12-, 24-, and 36-month CSS were 70.58% (95%CI: 66.66–74.50%), 72.12% (95%CI: 68.96–75.28%), and 71.12% (95%CI: 67.95–74.29%), demonstrating a quantitative method for predicting survival rates (Fig. 5). The calibration curves showed good agreement between the nomogram model and observation groups at 12-, 24-, and 36-month (Fig. 6). The nomogram was further proved to have appropriate clinical applicability.

## Discussion

SRCC is an exceedingly rare pathological type of colorectal cancer. Many patients with colorectal SRCC are diagnosed at an advanced stage due to their early atypical clinical presentations (Weng et al. 2022). And local and distant metastases are more frequently found in patients with SRCC, even at an early stage of the disease (Kim et al. 2021; Wu et al. 2021). However, most published studies about colorectal SRCC are small sample data or case reports due to the low incidence of colorectal SRCC (Weng et al. 2022; Korphaisarn et al. 2019; Tokunaga et al. 2019). A total of 38 patients were histologically confirmed as colorectal SRCC in Peng Wu’s study. Patients with SRCC were diagnosed with the worst CSS of all colorectal cancer patients, with 3-year and 5-year CSS rates of 19.3% and 9.2%, respectively (Wu et al. 2023). In addition, Nitsche U et al. extracted 28,056 colorectal patients (160 SRCC patients) from the Munich Cancer Registry. In SRCC patients, the 5-year CSS was reported to be approximately 59%, and locoregional and distant recurrence were more likely to occur. Primary tumor site, T/N/M stage, lymphatic/vascular invasion, and histological grade were independent risk factors of CSS (Nitsche et al. 2016). These articles have some limitations due to the limited sample size of SRCC patients. As a result, we incorporated extensive sample data from the SEER database to evaluate the CSS of SRCC patients.

In our study, a total of 1051 SRCC patients were included from the SEER database. More patients with T4 stage, N2 stage, tumor grade III, and grade IV were found in SRCC cases which were consistent with previous studies reporting that SRCC was often initially diagnosed at an advanced stage (Puccini et al. 2022; Attia et al. 2022). In addition, CSS at 12-, 24-, and 36-month were estimated to be 77.1% (95%CI: 74.6–79.7%), 58.6% (95%CI: 55.6–61.6%), and 48.6% (95%CI: 45.6–51.7%), respectively, among patients with SRCC and 80.9% (95%CI: 78.6–83.3%), 68.6% (95%CI: 65.8–71.4%), and 60.9% (95%CI: 58.0–64.0%) among those with adenocarcinoma. Patients with SRCC were associated with a worse prognosis than patients with adenocarcinoma. Similarly, SRCC was reported to be an independent prognostic risk factor for CSS of stage II and III colon cancer (HR: 4.2, 95%CI: 1.6–10.6, *P* = 0.003) (Qwaider et al. 2022). The poor prognosis of SRCC is closely related to the active invasion of the tumor and the high chance of lymph node and vascular metastasis (Kerckhoffs et al. 2020; Shu et al. 2018).

Age, T stage, N stage, and tumor size were identified as independent prognostic factors for survival according to the analysis of 1051 SRCC patients screened from the SEER database. A propensity score-matched analysis was performed on stage I and II colon cancer patients by Ackermann CJ et al. Tumor location, tumor stage, grade, age, gender, marital status, and CEA were identified as independent prognostic indicators for CSS (Ackermann et al. 2018). Similarly, Zhuang Z et al. analyzed 1675 stage II/III colorectal SRCC patients. They concluded that age < 65, tumor size ≤ 5 cm, AJCC stage II, radiotherapy, and chemotherapy were associated with better CSS in colorectal SRCC patients (Zhao et al. 2020). Studies have shown that tumor TNM stage was closely related to colorectal tumor prognosis (Mlecnik et al. 2023; Sun et al. 2022; Delattre et al. 2022). A total of 714 (67.5%) patients with the T3 stage, 259 (24.5%) patients with the N0 stage, 249 (23.6%) patients with the N1 stage, and 547 (51.8%) patients with the N2 stage were enrolled in the study. The T4 stage was associated with a deeper depth of tumor invasion than the T3 stage, and the N2 stage was associated with more lymph node metastases than N0/N1 stage. Colorectal mucosal invasion and lymph node metastasis are common pathways for metastasis and are crucial factors in determining the prognosis (Xu et al. 2022; Storli et al. 2014).

Regarding tumor size, tumor size < 40 mm showed a lower prognostic risk of colorectal SRCC, according to our findings. Osman MH performed a machine learning model for predicting postoperative 5-year survival of patients with colorectal cancer, demonstrating that tumor size had a high predictive value for survival prediction (Osman et al. 2022). Large tumor size is associated with advanced stage, more nodal invasion, and poorer differentiation (Alese et al. 2021; Tarazona et al. 2019). Six hundred and ninety-two patients with T1 colorectal cancer were identified between 2000 and 2016 from the SEER database. Multivariate analysis showed higher lymph node metastasis rates were more likely to be discovered in increasing tumor size (Ramai et al. 2021). According to the previous articles, the larger the tumor size, the worse the prognosis of the patients with colorectal SRCC (Jung et al. 2021; Burzykowski et al. 2019).

Moreover, SRCC patients with age < 60 were more likely to have better CSS. Younger patients with colorectal cancer were reported that they had a higher 5-year survival rate. Paradoxically, younger patients tended to have lower survival in Sifaki-Pistolla D’ study. They noticed that colorectal cancer is often diagnosed at an advanced stage in younger patients (Sifaki-Pistolla et al. 2022). Further studies are needed to determine the impact of early-onset colorectal cancer on survival.

A prognostic nomogram was constructed based on T/N stage, age, and tumor size in this study to predict the CSS of SRCC patients, which transformed the Cox regression results into a visual graph and made the results of the prediction model more readable and convenient for the CSS evaluation of SRCC patients. Previous studies have constructed nomograms to predict the prognosis of colorectal SRCC. Diao JD et al. and Kou FR et al. evaluated 3- and 5-year overall survival by creating nomograms (Diao et al. 2021; Kou et al. 2021), but limitations existed in these studies. Data bias and confounding variables existed in these studies, inevitably affecting the results. In addition, in the Diao JD study, the overall survival of colorectal SRCC was evaluated instead of CSS. Our nomogram can help clinicians assess the survival of colorectal SRCC.

Several limitations existed in our study. Firstly, there were some inherent flaws that could have been improved in the observational analysis of the SEER database. Although this study evaluated the impact of postoperative chemotherapy on patient survival, chemotherapy cycles and regimens were lacking. Secondly, this study did not include some clinicopathological variables, such as CA199 level, neoadjuvant therapy, distant metastasis, and radiotherapy, and may be critical for patient survival. Thirdly, specific details regarding tumor pathology, such as microsatellite stability, BRAF, and K-RAS, were unavailable in the SEER database. Finally, using the 6th edition of the AJCC TNM staging system to assess primary pathological tumor T and N stages meant that patients with colorectal SRCC staging by other AJCC stages were not included in the study, which inevitably led to selection bias.

## Conclusion

In conclusion, patients with colorectal SRCC have a worse prognosis than those with colorectal adenocarcinoma. And chemotherapy, T3/N0 stage, grade I-II, and small tumor size may help improve survival rate. In addition, a nomogram was constructed to predict 12-, 24-, and 36-month CSS for colorectal SRCC based on age, T stage, N stage, and tumor size, which would help clinicians to evaluate the prognosis of patients with SRCC.

## Data Availability

The datasets generated during and/or analyzed during the current study are available from the corresponding author on reasonable request.
